# Comparison of ChatGPT-4o and DeepSeek R1 in the Management of Ophthalmological Emergencies—An Analysis of Ten Fictional Case Vignettes

**DOI:** 10.3390/jcm14248927

**Published:** 2025-12-17

**Authors:** Dominik Knebel, Siegfried Priglinger, Benedikt Schworm

**Affiliations:** Department of Ophthalmology, University Hospital, LMU Munich, Mathildenstraße 8, 80336 Munich, Germany

**Keywords:** ChatGPT-4o, DeepSeek R1, artificial intelligence, AI, emergencies, triage

## Abstract

**Background:** Generative artificial intelligence (AI) applications have gained increasing popularity in recent years and are used by an ever-increasing number of people on a day-to-day basis. While the performance of the earlier-generation generative AI ChatGPT-3.5 in the context of ophthalmologic emergencies has been previously assessed, the purpose of this study is to analyze the performance of the newer-generation generative AIs DeepSeek R1 (Hangzhou DeepSeek Artificial Intelligence Co., Ltd., Hangzhou, China) and ChatGPT-4o (OpenAI Inc., San Francisco, CA, USA) in the context of diagnosis, triage and prehospital management of ophthalmological emergencies. **Methods:** Ten previously published fictional case vignettes representing queries in the English language of patients experiencing acute ophthalmological symptoms were entered into the generative AIs DeepSeek R1 and ChatGPT-4o. The interaction with the generative AIs followed a previously described structured interaction path. In a random order, each case vignette was entered into separate chats five times, producing a total of 50 answers from each generative AI. Each answer was analyzed according to a previously published manual. **Results:** We observed better values for DeepSeek R1 compared to ChatGPT-4o in terms of treatment accuracy (60% compared to 50%), the share of answers containing wrong (46% compared to 60%) or conflicting information (30% compared to 40%), the share of answers that correctly captured the overall severity of symptoms (98% compared to 78%), as well as the share of potentially harmful answers (38% compared to 50%). Moreover, DeepSeek R1 more frequently provided a single diagnosis (20% compared to 16%) and specific treatment advice (42% compared to 20%) than ChatGPT-4o. Both generative AIs showed a diagnostic accuracy of 100%, i.e., whenever they provided a single diagnosis, this was indeed the correct diagnosis. In terms of triage accuracy, ChatGPT-4o performed slightly better than DeepSeek R1 (73% compared to 66%). In contrast to DeepSeek R1, which never directed questions back at the user, ChatGPT-4o always did. The direction of questions at the user enables dialogues with ChatGPT-4o that more closely resemble the actual taking of a patient’s history. However, DeepSeek R1 seems to perform better compared to ChatGPT-4o in terms of several important content-related metrics and has been shown to be more cost-effective in other studies. **Conclusions:** Both newer-generation generative AIs constitute remarkable milestones in the development of generative artificial intelligence. However, since potentially harmful recommendations were observed with both models, we currently do not recommend their use as sole source of information on ophthalmological emergencies for laypersons.

## 1. Introduction

Two years after the introduction of the generative artificial intelligence (AI) application Chat Generative Pre-Trained Transformer (ChatGPT) to the public by OpenAI LP (San Francisco, CA, USA), the generative AI DeepSeek R1 (Hangzhou DeepSeek Artificial Intelligence Co., Ltd., Hangzhou, China) has made headlines for its highly cost-efficient development as well as its performance, representing a serious alternative to the current models of ChatGPT, including ChatGPT-4o.

Most generative AI applications have not been designed for medical purposes [1]. However, DeepSeek R1 has reportedly already been deployed in the Chinese health care system shortly after its launch [2] and has been shown to be more cost-efficient than ChatGPT-o1 while performing at a comparable level in the ophthalmological context [3].

Beyond their possible deployment in health care institutions, an important consideration regarding the health impact of publicly available generative AI tools is their possible use by laypersons experiencing acute symptoms. In 2025, OpenAI estimates the number of weekly users of ChatGPT at 700 million and reports that 49% of queries were users asking for advice (as opposed to 40% of queries delegating generative tasks and 11% expressive tasks or play) and that 70% of the usage was non-work-related [4]. As early as in 2023, a cross-sectional survey conducted among people who used ChatGPT at least monthly, 78.4% of respondents reported that they were willing to use ChatGPT for self-diagnosis [5]. In a recent representative survey among 1145 persons in Germany aged 16 and older, 45% of respondents stated that they currently consult generative AI for self-diagnosis or general health queries, 55% stated that they trust the responses of the generative AI, 30% stated that they value them like a second opinion obtained by a physician, and 16% reported having already disregarded advice from their physician in favor of the advice given by the AI [6]. However, AI-generated content can be error-prone, and delayed diagnosis linked to ChatGPT use for self-diagnosis has been reported [7].

In 2023, our group assessed the performance of ChatGPT-3.5 in the context of ophthalmological emergencies using ten custom-made short clinical case vignettes and a standardized evaluation protocol [8]. While observed triage accuracy was comparable to ophthalmological staff triaging via telephone, we found potentially harmful answers in 32% of responses and therefore concluded that laypersons should not rely on ChatGPT-3.5 as a primary source of information on ophthalmological emergencies [8]. Subsequently, its successor ChatGPT-4 has been extensively assessed in terms of ophthalmological triage [9,10,11]. One study analyzed actual de-identified electronic patient queries and tested triage by ChatGPT-4 versus ophthalmology residents. The authors observed more urgent scheduling and more subspecialist referrals by ChatGPT-4 while at the same time observing instances of potential patient harm through delayed scheduling by ChatGPT-4 [9]. However, in another study ChatGPT-4, with a triage accuracy of 98%, outperformed not only Bing Chat (84%) but also ophthalmology residents (96%) [10]. In another study, it outperformed Google Bard with a triage accuracy of 96% vs. 84% [11]. A Swiss-based study group showed that the performance of ChatGPT-4 in ophthalmologic triage can be further enhanced by fine-tuning of the model, observing a high agreement between the fine-tuned ChatGPT-4 and three ophthalmologists (Cohen’s κ 0.79) [12].

Recently, several studies have evaluated ChatGPT-4o and DeepSeek R1 in the ophthalmologic domain: In a recent preprint, both ChatGPT-4o and DeepSeek R1 have been reported to outperform ophthalmologists on 11 glaucoma cases [13]. Another study found Qwen2.5 and DeepSeek R1 to outperform ChatGPT-4o in terms of ophthalmologic queries [14], while in another study DeepSeek outperformed ChatGPT-4o in terms of accuracy answering myopia-related questions [15].

While these evolutions seem promising, the outperformance of newer over older models is not consistently observed throughout the literature: A recent study compared the newer model ChatGPT-4o to ChatGPT-3.5 with regard to the European Board of Ophthalmology Diploma (EBOD) examination and found no clear advantage of the newer model over the older: ChatGPT-4o outperformed ChatGPT-3.5 on the easier questions, while underperforming on the more difficult questions [16]. Similarly, in a study comparing ChatGPT-4o, Gemini, and ChatGPT-3.5 in terms of accuracy when answering questions related to thyroid eye disease, the newer ChatGPT-4o and Gemini were outperformed by the older ChatGPT-3.5 [17]. Moreover, another recent study compared different generative AI models, including DeepSeek R1 and ChatGPT-4o, with regard to the preferred practice patterns of the American Academy of Ophthalmology, reporting frequent discordant answers and potentially harmful answers by all models [18]. The features and capabilities of publicly available generative AIs have been increasing ever since the release of ChatGPT-3.5. However, as evidenced by the aforementioned studies [16,17,18], it is not guaranteed whether these greater capabilities actually translate into better content-related parameters, such as accuracy or potential for harm. In the setting of ophthalmologic emergencies, however, the user’s subjective experience of general improvements in the AI’s features might lead to an overestimation of its trustworthiness.

The purpose of this study is (1) to compare the newer-generation DeepSeek R1 to ChatGPT-4o in the context of ophthalmological emergencies, exploring differences between both models, and (2) to assess how these results compare to the performance of ChatGPT-3.5 in our prior analysis [8].

## 2. Materials and Methods

### 2.1. Study Design

Since our assessment of ChatGPT-3.5, a large number of medical research articles involving generative AI have been published, and scientific methods have undergone further development. This includes, for example, the refinement of prompting methods to reach better outcomes across a variety of tasks [19], as well as the development of standardized evaluation tools [20]. However, we chose to adhere to the methodology of our prior analysis of ChatGPT-3.5 [8] for the following reasons:

1. Our study was designed with possible layperson use of publicly available generative AI tools for self-diagnosis and self-triage in mind. In most countries, self-reported AI competence is quite low, and the majority of the general public did not undergo any specific AI training [21]. Hence, knowledge of advanced prompting methods cannot be assumed of the general public, and more valid results may be obtained by adhering to our simple prompting protocol that was specifically designed in short and simple language to resemble laypersons’ requests.
2. Standardized evaluation tools such as the CLEAR tool are designed to enable comparable evaluation of AI outputs across a wide range of tasks. It comprises grading of the AI-generated answers according to five different items (sufficiency, accuracy (lack of false information), evidence support, clarity/understandability, and relevance (lack of irrelevant information)) on a five-point Likert scale ranging from excellent to poor [20]. While there is some overlap with our own evaluation protocol, the latter is tailored to the evaluation in the context of triage of emergencies. It provides certain evaluation parameters that are critical in this specific context, but are not comprised in tools such as CLEAR (e.g., potential for harm).
3. While perfect comparability to our prior analysis of ChatGPT-3.5 cannot be reached due to the involvement of metachronous qualitative evaluations, we strive for the best possible comparability by following the same methodology.

### 2.2. Case Vignettes

We used ten custom-made fictional case vignettes (Hordeolum, Pediatric Leukocoria, Flashes and floaters, Sudden monocular vision loss, Sudden and painful monocular vision loss, Sudden onset diplopia, Dry eye, Monocular red eye, Corneal erosion, Alkali burn) that were developed by our group and have been previously published. They are conceptualized as simple, short, and stereotypic English sentences, in order to mimic a laypersons’ description of acute ocular symptoms. The specific symptoms were selected to cover a wide variety of symptoms from different ophthalmologic subspecialties, as well as a wide spectrum across different levels of urgency and severity.

### 2.3. Models

We used the free versions of DeepSeek R1 (Hangzhou DeepSeek Artificial Intelligence Co., Ltd., Hangzhou, China) and ChatGPT-4o (OpenAI Inc., San Francisco, CA, USA) in their standard settings, i.e., without any adjustments or customization to best simulate layperson use.

### 2.4. Prompting

We followed a standardized prompting protocol using a previously published interaction-pathway. The first prompt consisted of a presentation of the vignette and a question about diagnosis and treatment. If visiting a physician was recommended in the answer to the first prompt (answer 1), the second prompt asked for the urgency of that consultation. Otherwise, the second prompt asked the generative AI to confirm whether consultation with a physician is necessary. A third prompt could be used for clarification or further inquiry at the investigator’s discretion after the answer to the second prompt (answer 2).

During data acquisition, it became clear that the responses given by ChatGPT-4o contained questions directed back at the user, whereas the responses given by DeepSeek R1 did not. Clearly, exploration of the resulting possibilities of interactional dialogues with ChatGPT-4o would constitute an interesting topic on its own. However, for the sake of structural clarity and standardization, as well as for better comparability with both DeepSeek R1 and the results of our prior analysis of ChatGPT-3.5, we chose not to explore the interactional possibilities of ChatGPT-4o for this analysis: Questions directed back at the user were left unanswered, and their presence or absence in answer 1 did not change the fact that the second prompt was strictly chosen out of two possible templates depending on whether visiting a physician was recommended in answer 1. The AI’s calculation of a response to a prompt has probabilistic features. Hence, if the same prompt is entered twice (even in separate dialogues), the responses will differ from each other. Therefore, repetition is necessary to enhance the robustness of results. In our prior study, we adopted the protocol of using five repetitions from other early studies on ChatGPT-3.5. In this analysis, we adhere to the methodology of our prior study for comparability reasons. Hence, the aforementioned standardized prompting protocol was repeated a total of five times for each combination of case vignette and generative AI, yielding a total of 100 responses, including 50 responses by DeepSeek R1 and ChatGPT-4o, respectively. The different vignettes were presented in the same random order to DeepSeek R1 and ChatGPT-4o, and each vignette was presented in a new dialogue.

### 2.5. Evaluation of Responses

All responses were analyzed according to a previously published standardized manual. Both generative AIs were compared on a global and vignette level to each other with regard to 17 items (Diagnostic specificity, Number of differential diagnoses, Diagnostic accuracy, Treatment specificity, Treatment accuracy, Unconditional advice to consult a physician in answer 1, Disclaimer, Unconditional advice to consult a physician in answer 2, Provision of urgency information, Triage accuracy, Provision of recommended pre-hospital measures, Appropriateness of recommended pre-hospital measures (APM), Direction of questions back at the user, Wrong information, Conflicting information, Overall accuracy of reflection of symptom severity, and Overall potential to inflict harm).

Triage accuracy was checked against predefined and previously published urgency levels assigned to each vignette. Those urgency levels were attained by consensus of the authors of our prior study. As not all vignettes allow narrowing down the differential diagnoses to one single diagnosis, and as the information provided in the AI responses varied widely, including many different differential diagnoses, treatment options, and other factoids, we could not rely on predefined ground truths for evaluation of Diagnostic accuracy, Treatment accuracy, APM, Wrong information, Conflicting information, Overall accuracy of reflection of symptoms, and Overall potential to inflict harm. These measures were therefore evaluated by the authors’ judgment. If unconventional factoids were presented in the AI-generated responses, their truth was checked via research with appropriate resources (e.g., PubMed research).

During evaluation, emphasis was placed on careful distinction between wrong information, conflicting information, and potentially harmful information. This is well illustrated by the answers of ChatGPT-4o to vignette F (sudden-onset diplopia), which gave advice on how to distinguish monocular from binocular diplopia. While this process was inaccurately represented several times (wrong information), the answers differed in terms of whether the AI contradicted itself throughout the response (conflicting information). Likewise, one of the instances was judged as potentially harmful because it may lead to the user underestimating the urgency of their actually binocular diplopia (overall potential to inflict harm), while another instance has been judged as without potential for harm, as it would lead to the user misdiagnosing their monocular diplopia as binocular diplopia and therefore overestimating urgency (no overall potential to inflict harm).

While the Number of differential diagnoses was processed as a rationally scaled parameter, and four items were analyzed on ordinal scales (Diagnostic specificity, Treatment specificity, APM, and Overall accuracy of reflection of symptom severity), the other items were processed on a binary/nominal scale (yes/no).

The ordinal scales were defined as follows: Diagnostic specificity = 1 was defined as the provision of a single diagnosis (independent of the actual accuracy of this diagnosis). The next lower levels were defined as provision of a single most probable diagnosis among a set of possible differential diagnoses (diagnostic specificity = 2), provision of a set of possible differential diagnoses without specification of a most probable diagnosis (diagnostic specificity = 3), and no provision of any diagnosis at all (diagnostic specificity = 4).

Treatment specificity = 1 was defined as the provision of specific treatment advice (e.g., “Treatment X is recommended for your condition”). The lower levels were defined as general information on treatment (e.g., “Treatment for differential diagnosis A would be X, treatment for differential diagnosis B would be Y, …”, treatment specificity = 2), vague information on treatment (e.g., “Treatment may include medication or surgery”, treatment specificity = 3), and the lack of information on treatment (treatment specificity = 4).

Overall accuracy of reflection of symptom severity was judged as appropriate (value = 1), overestimation of severity (value = 2), or underestimation of severity (value =3). An illustrative example is given by the different responses of DeepSeek R1 to vignette B (pediatric leukocoria): One response listed retinoblastoma first in the list of the most common causes (judged as overestimation of severity, since retinoblastoma generally does not constitute the most common differential diagnosis of pediatric leukocoria). Other responses presented retinoblastoma as the most feared but not the most likely differential diagnosis and were therefore judged as appropriately reflecting the severity. One response implied that the condition was less urgent than stated by our predefined urgency level and was therefore judged as an underestimation of severity.

In terms of information on pre-hospital measures, for any response containing potentially harmful information or lacking the absolutely crucial pre-hospital measure of immediately starting to rinse the eye in the case of an alkali burn, APM was defined as 0. Examples observed in this analysis or in our prior study include the recommendation of self-medication for acute, painless vision loss (ChatGPT-4o) and the lack of the crucial advice to start immediately rinsing the eye in the case of an alkali burn (ChatGPT-3.5). The responses that were not graded as APM = 0 were graded as APM = 1 if they contained conflicting advice on pre-hospital measures. An example of information judged as conflicting, but not harmful includes a response to vignette B (Pediatric leukocoria) that included not only the advice to avoid bright lights and photos with flash, but also the contrary advice to take photos with flash in order to document the leukocoria (ChatGPT-4o). APM = 2 was defined as provision of only useless (but not harmful or conflicting) information, APM = 3 was defined as provision of useful as well as useless (but not harmful or conflicting) information, and APM = 4 was defined as the provision of only useful (and not harmful or conflicting) information. An observed example of a recommendation judged useless (but not harmful) was to keep the eye and surrounding area clean to prevent secondary infection (DeepSeek R1) for vignette B (Pediatric leukocoria). An observed example of a recommendation judged useful was patching one eye to alleviate sudden binocular diplopia and avoidance of driving oneself while not delaying an immediate visit to the emergency room (DeepSeek R1).

### 2.6. Statistical Analysis

A descriptive statistical analysis was performed using Microsoft Excel (Microsoft Corporation, Redmond, WA, USA) on the vignette level as well as global level. On a global level, explorative statistical analysis was performed using SPSS Statistics Version 29 (IBM Corporation, Armonk, NY, USA). Binary parameters were analyzed using a Chi^2^ test or Fisher’s exact test, ordinally scaled and non-normally distributed parameters were analyzed using a Mann–Whitney U test, and normally distributed parameters were analyzed using a *t*-test. Shapiro–Wilk and Kolmogorov–Smirnov tests were used to establish normality. Due to the explorative character of the statistical analysis, a local α = 0.05 was considered statistically significant. We used regression analysis to test whether for a given vignette, the share of answers containing a disclaimer stating that the AI cannot give a diagnosis was correlated with the share of potentially harmful answers for the respective vignette.

## 3. Results

### 3.1. Global Results

**Figure 1:**
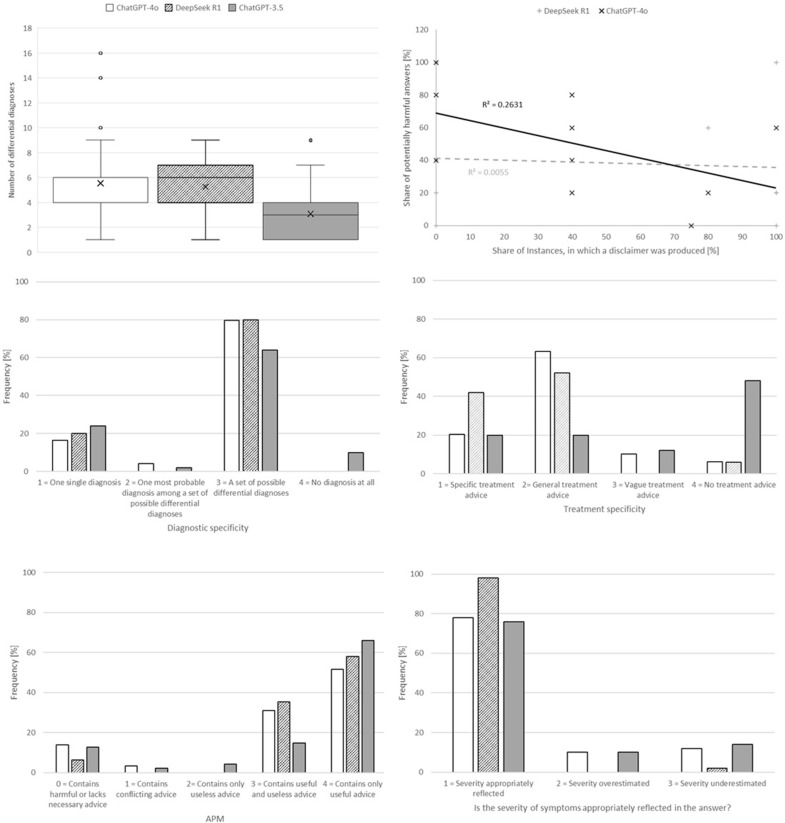
Number of differential diagnoses, Diagnostic and Treatment specificity, Appropriateness of recommended pre-hospital measures (APM), as well as Overall accuracy of reflection of symptom severity for ChatGPT-4o, DeepSeek R1, and ChatGPT-3.5 (reproduced from our prior publication) [8]. First row, right: The share of answers per vignette that contain an explicit disclaimer warning that the model cannot give a diagnosis or medical advice, and its correlation with the share of potentially harmful answers per vignette.

The global results are presented in Table 1 and Figure 1. For comparison, the corresponding data for ChatGPT-3.5 have been reproduced from our prior publication [8]. For the number of differential diagnoses, both Shapiro–Wilk- and Kolmogorov–Smirnov tests rejected normality; hence, a Mann–Whitney U test was used to compare ChatGPT-4o and DeepSeek R1 with regard to this parameter.

Statistically significant differences between ChatGPT-4o and DeepSeek R1 were observed with regard to four parameters (Direction of questions back at the user, Treatment specificity, Overall accuracy of reflection of symptom severity, and Disclaimer). While ChatGPT-4o directed questions back at the user in every instance, DeepSeek R1 never did. The questions directed at the user included whether help was needed to find a care provider, as well as questions aimed at clarification of the patient’s history. In several instances, ChatGPT-4o asked the user to provide a photo of the affected eye. However, DeepSeek R1 provided more specific treatment advice, its answers better reflected the symptom severity, and it more frequently provided a disclaimer stating that it cannot give a medical diagnosis.

While the observed differences between ChatGPT-4o and DeepSeek R1 with regard to the other parameters were not statistically significant, a tendency towards better values for DeepSeek R1 compared to ChatGPT-4o was observed in terms of the parameters Treatment accuracy, the share of answers containing wrong or conflicting information, as well as the share of answers harboring a potential for harm. However, for Triage accuracy, a better value was observed for ChatGPT-4o (albeit not statistically significant).

### 3.2. Disclaimer and Vignette-Level Results

The vignette-level results for ChatGPT-4o and DeepSeek R1 are presented in Table 2 and Table 3, respectively. The share of potentially harmful answers for each vignette varies considerably between the different vignettes.

This share of potentially harmful answers can be regarded as a proxy for the risk a user would face when relying on the specific model as a primary data source on acute symptoms corresponding to the vignette. In a real-world setting, users might unconsciously estimate this risk based on the answer they retrieved from the AI and accordingly decide whether or not to consult other sources of information. In many instances, the AI-generated responses contain disclaimers stating that the AI is not a physician, cannot give a diagnosis, or similarly emphasize its limitations. The presence of such a disclaimer may have an effect on the users’ risk estimates, as they are reminded that they are using the AI out of its scope.

These considerations lead to the question of whether the presence of such a disclaimer is indeed correlated with a higher risk (i.e., a higher share of potentially harmful answers). Figure 1 (first row, right) shows the share of potentially harmful answers plotted against the share of answers containing a disclaimer. For DeepSeek R1, there is nearly no linear correlation between the two parameters (i.e., the presence of such a disclaimer does not indicate a higher risk). For ChatGPT-4o, there is a weak negative linear correlation, suggesting the absence of a disclaimer as a weak indicator of a higher potential for harm (i.e., not the presence, but the absence of a disclaimer indicates higher risk).

## 4. Discussion

Compared to our first analysis of ChatGPT-3.5 in early 2023, the most striking difference is that ChatGPT-4o directed questions back at the user in every instance, thus more closely resembling the taking of a patient’s history by medical professionals. In contrast, DeepSeek R1 did not direct any questions back at the user.

However, this difference between the models could potentially be misleading towards the user, generating the impression of higher trustworthiness of ChatGPT’s answers compared to DeepSeek. In reality, the content-related metrics analyzed in this study did not show any signs of outperformance of ChatGPT. To the contrary, DeepSeek R1 gave significantly more specific treatment advice, and its answers more accurately reflected the severity of symptoms.

Moreover, in accordance with the findings of Maino and colleagues in terms of EBOD examination questions [16] and the findings of Bajar and colleagues in terms of queries related to thyroid–eye disease [17], our data do not show consistent overperformance of ChatGPT-4o compared to ChatGPT-3.5 as evaluated in our prior analysis [8].

We observed a lower share of harmful answers from DeepSeek R1 compared to ChatGPT-4o. Albeit not statistically significant, this might be a clinically relevant tendency, especially when considering the small sample size that enables only large effect sizes to be detected with high power.

Our observation of a lower share of harmful answers from DeepSeek R1 contrasts the findings from Taloni and colleagues showing a higher share of harmful answers from DeepSeek R1 [18]. Moreover, we found a much higher share of potentially harmful answers compared to the study by Taloni and colleagues, which might be due to methodological differences or differences in the type and complexity of questions presented to the models [18]. Important methodological differences include public availability of the source of the questions as well as the ground truth against which the answers are compared. As Taloni and colleagues derived their questions and their ground truth from the publicly available Preferred Practice Patterns of the American Academy of Ophthalmology (AAO PPP), those data might have been included in the training sets of the investigated AIs. In contrast, we did not establish ground truths other than expected diagnoses and urgency levels. While those, of course, have been previously published and therefore might possibly be included in the training sets, a large fraction of what comprises the overall potential for harm in our investigation does not correspond to any predetermined ground truth, but was fact-checked manually by the authors. Beyond the availability of ground truths, triage, diagnosis, and recommendation of treatment is a far more complex task than answering a specific question for which the answer can be supported with published evidence (i.e., the AAO PPP).

As in our prior analysis of ChatGPT-3.5, the presence of a disclaimer was not an indication for a higher risk of harm (for both ChatGPT-4o and DeepSeek R1). To the contrary, for ChatGPT-4o, the absence of a disclaimer could be seen as a weak indicator of a higher potential for harm. For DeepSeek R1, no correlation between the disclaimer and the potential for harm was found. However, DeepSeek R1 significantly more frequently produced such a disclaimer compared to ChatGPT-4o, reminding the user that they should not use the model to obtain a diagnosis.

The limitations of our methodology have been extensively discussed in our prior publication [8]. They include potential differences between our carefully worded case vignettes and real patient queries, as well as the methodology being designed primarily to evaluate triage accuracy, APM, and potential for harm, and not diagnostic or treatment accuracy. While we adopted the same methodology as in our prior study, comparability between both assessments might be reduced due to the metachronous qualitative evaluations. This is the reason we did not include the results of our prior study in the explorative statistical analysis. Moreover, in the context of ChatGPT-4o directing questions at the user, we did not further explore and analyze the potential interactions opening up when supplying further information by answering those questions. Continued interaction with ChatGPT-4o could potentially yield better results in terms of diagnostic and treatment accuracy and specificity.

Both ChatGPT-4o and DeepSeek R1 constitute milestones within the ongoing evolution of generative AI. The direction of questions at the user by ChatGPT-4o opens up the possibility of dialogues that more closely mirror the actual taking of a patient’s history by medical professionals. DeepSeek R1, on the other hand, which has been associated with better cost-efficiency in a recent study, seems to outperform ChatGPT-4o in terms of treatment specificity and in terms of how well the answers reflect the severity of the presented symptoms. However, since we observed potentially harmful recommendations by both models, which is in line with the literature, we currently do not recommend their use by laypersons as the sole source of information on ophthalmological emergencies.

## Figures and Tables

**Table 1:** Performance of ChatGPT-4o compared to DeepSeek R1. The values for ChatGPT-3.5 are reproduced from our prior publication. APM = Appropriateness of recommended pre-hospital measures. Significant *p*-values are highlighted in bold print. The ordinal scales for Diagnostic specificity, Treatment specificity, APM, and Overall reflection of the severity of presented symptoms can be found in Figure 1. *: The same data points were observed for both ChatGPT-4o and DeepSeek R1, hence provision of a *p*-value is not useful.

Item	ChatGPT-4o	DeepSeek R1	*p*-Value	ChatGPT-3.5
Diagnostic specificity (median, [range])	3, [1, 3]	3, [1, 3]	0.48	3, [1, 4]
Number of differential diagnoses (median, [range])	6, [1, 16]	6, [1, 9]	0.219	3, [1, 9]
Diagnostic accuracy (%)	100	100	n/a *	62
Treatment specificity (median, [range])	2, [1, 4]	2, [1, 4]	**0.007**	3, [1, 4]
Treatment accuracy (%)	50	60	0.52	100
Unconditional recommendation to consult a physician in answer 1 (%)	61	64	0.388	94
Disclaimer (%)	41	58	**0.044**	62
Unconditional recommendation to consult a physician in answer 2 (%)	16	39	0.056	33
Information on urgency (%)	100	100	n/a *	100
Triage accuracy (%)	73	66	0.256	87
Pre-hospital measures recommended (%)	97	97	n/a *	100
APM (median, [range])	4, [0, 4]	4 [0,4]	0.223	4, [0, 4]
Questions directed at user (%)	100	0	**<0.001**	0
Wrong information (%)	60	46	0.115	24
Conflicting information (%)	40	30	0.148	36
Overall reflection of the severity of presented symptoms (median, [range])	1, [1, 3]	1, [1, 3]	**0.001**	1, [1, 3]
Harmful answers (%)	50	38	0.114	32

**Table 2:** Performance of ChatGPT-4o at the individual vignette level. APM = Appropriateness of recommended pre-hospital measures. *: Diagnostic accuracy is not well defined, since all responses to the corresponding vignette contained no diagnosis at all or only a set of differential diagnoses without specification of a single most probable diagnosis. **: Triage accuracy and APM are not well defined, since none of the responses to the corresponding vignette include an unconditional recommendation to consult a physician.

	Vignette Title	Diagnostic Accuracy	Disclaimer Contained	Triage Accuracy	APM (Median, [Range])	Potentially Harmful Answers
A	Hordeolum	- *	3/4 (75%)	- **	- **	0/5 (0%)
B	Pediatric leukocoria	- *	0/5 (0%)	4/5 (80%)	3, [1; 4]	2/5 (40%)
C	Flashes and floaters	- *	2/5 (40%)	1/5 (20%)	4, [4; 4]	4/5 (80%)
D	Sudden monocular vision loss	- *	2/5 (40%)	5/5 (100%)	3, [0; 4]	3/5 (60%)
E	Sudden, painful monocular vision loss	- *	2/5 (40%)	5/5 (100%)	3, [0; 3]	2/5 (40%)
F	Sudden onset diplopia	- *	0/5 (0%)	1/4 (25%)	4, [3; 4]	4/5 (80%)
G	Dry eye	2/2 (100%)	4/5 (80%)	- **	- **	1/5 (20%)
H	Monocular red eye	- *	5/5 (100%)	- **	- **	3/5 (60%)
I	Corneal erosion	5/5 (100%)	0/5 (0%)	1/1 (100%)	4, [4; 4]	5/5 (100%)
J	Alkali burns	3/3 (100%)	2/5 (40%)	5/5 (100%)	4, [0; 4]	1/5 (20%)

**Table 3:** Performance of DeepSeek R1 at the individual vignette-level. APM = Appropriateness of recommended pre-hospital measures. *: Diagnostic accuracy is not well defined, since all responses to the corresponding vignette contained no diagnosis at all or only a set of differential diagnoses without specification of a single most probable diagnosis. **: Triage accuracy and APM are not well defined, since none of the responses to the corresponding vignette include an unconditional recommendation to consult a physician.

	Vignette Title	Diagnostic Accuracy	Disclaimer Contained	Triage Accuracy	APM (Median, [Range])	Potentially Harmful Answers
A	Hordeolum	- *	0/5 (0%)	- **	- **	0/5 (0%)
B	Pediatric leukocoria	- *	0/5 (0%)	5/5 (100%)	4, [0; 4]	1/5 (20%)
C	Flashes and floaters	- *	4/5 (80%)	2/5 (40%)	3, [3; 3]	3/5 (60%)
D	Sudden monocular vision loss	- *	5/5 (100%)	4/5 (80%)	4, [3; 4]	1/5 (20%)
E	Sudden, painful monocular vision loss	- *	5/5 (100%)	5/5 (100%)	4, [3; 4]	1/5 (20%)
F	Sudden onset diplopia	- *	0/5 (0%)	0/5 (0%)	3.5, [3; 4]	5/5 (100%)
G	Dry eye	- *	5/5 (100%)	- **	- **	0/5 (0%)
H	Monocular red eye	- *	5/5 (100%)	1/1 (100%)	4, [4; 4]	1/5 (20%)
I	Corneal erosion	5/5 (100%)	5/5 (100%)	1/1 (100%)	0, [0; 0]	5/5 (100%)
J	Alkali burn	5/5 (100%)	0/5 (0%)	3/5 (60%)	4, [4; 4]	2/5 (40%)

## Data Availability

The data presented in this study are available in this article.
